# Supplementary material for: Modeling glioblastoma heterogeneity as a dynamic network of cell states
Source: Mol Syst Biol. 2021 Sep 16;17(9):e10105. doi: 10.15252/msb.202010105 (PMC8444284; doi:10.15252/msb.202010105)
Supplement: Supplementary file 5 — Source Data for Figure 3 [file MSB-17-e10105-s001.zip › Figure3A_sourcedata/GSEA_3065/hallmarks_state5.GseaPreranked.1623416302988/HALLMARK_REACTIVE_OXYGEN_SPECIES_PATHWAY.html]

Details for gene set HALLMARK\_REACTIVE\_OXYGEN\_SPECIES\_PATHWAY[GSEA]

|  || Dataset | state5 |
| Phenotype | NoPhenotypeAvailable |
| Upregulated in class | na\_neg |
| GeneSet | HALLMARK\_REACTIVE\_OXYGEN\_SPECIES\_PATHWAY |
| Enrichment Score (ES) | -0.640178 |
| Normalized Enrichment Score (NES) | -1.8919921 |
| Nominal p-value | 0.0 |
| FDR q-value | 2.5E-4 |
| FWER p-Value | 0.001 |
Table: GSEA Results Summary

  

Fig 1: Enrichment plot: HALLMARK\_REACTIVE\_OXYGEN\_SPECIES\_PATHWAY      
 Profile of the Running ES Score & Positions of GeneSet Members on the Rank Ordered List

  

| PROBE | GENE SYMBOL | GENE\_TITLE | RANK IN GENE LIST | RANK METRIC SCORE | RUNNING ES | CORE ENRICHMENT || 1 | PRDX4 |  |  | 80 | 0.553 | 0.0355 | No |
| 2 | SELENOS |  |  | 197 | 0.441 | 0.0580 | No |
| 3 | PRNP |  |  | 851 | 0.232 | 0.0037 | No |
| 4 | SCAF4 |  |  | 1334 | 0.168 | -0.0367 | No |
| 5 | ERCC2 |  |  | 3674 | 0.021 | -0.2964 | No |
| 6 | SBNO2 |  |  | 3760 | 0.017 | -0.3046 | No |
| 7 | TXNRD2 |  |  | 4343 | -0.008 | -0.3690 | No |
| 8 | OXSR1 |  |  | 4502 | -0.014 | -0.3855 | No |
| 9 | MSRA |  |  | 5245 | -0.042 | -0.4650 | No |
| 10 | CAT |  |  | 5358 | -0.047 | -0.4738 | No |
| 11 | GCLM |  |  | 5728 | -0.063 | -0.5100 | No |
| 12 | GSR |  |  | 5778 | -0.065 | -0.5102 | No |
| 13 | NDUFS2 |  |  | 5820 | -0.066 | -0.5095 | No |
| 14 | JUNB |  |  | 6665 | -0.112 | -0.5948 | No |
| 15 | GLRX |  |  | 6822 | -0.123 | -0.6024 | No |
| 16 | PFKP |  |  | 6845 | -0.124 | -0.5949 | No |
| 17 | STK25 |  |  | 6957 | -0.133 | -0.5966 | No |
| 18 | SOD2 |  |  | 6961 | -0.134 | -0.5862 | No |
| 19 | PTPA |  |  | 7271 | -0.164 | -0.6075 | No |
| 20 | GLRX2 |  |  | 7428 | -0.185 | -0.6101 | No |
| 21 | PDLIM1 |  |  | 7628 | -0.208 | -0.6157 | Yes |
| 22 | CDKN2D |  |  | 7721 | -0.222 | -0.6081 | Yes |
| 23 | HMOX2 |  |  | 7729 | -0.223 | -0.5910 | Yes |
| 24 | TXNRD1 |  |  | 7767 | -0.228 | -0.5769 | Yes |
| 25 | G6PD |  |  | 8271 | -0.346 | -0.6053 | Yes |
| 26 | PRDX1 |  |  | 8584 | -0.476 | -0.6019 | Yes |
| 27 | NQO1 |  |  | 8588 | -0.479 | -0.5638 | Yes |
| 28 | PRDX6 |  |  | 8673 | -0.540 | -0.5298 | Yes |
| 29 | NDUFA6 |  |  | 8687 | -0.550 | -0.4870 | Yes |
| 30 | PRDX2 |  |  | 8749 | -0.611 | -0.4447 | Yes |
| 31 | LAMTOR5 |  |  | 8806 | -0.656 | -0.3983 | Yes |
| 32 | NDUFB4 |  |  | 8810 | -0.659 | -0.3457 | Yes |
| 33 | SOD1 |  |  | 8848 | -0.709 | -0.2929 | Yes |
| 34 | ATOX1 |  |  | 8851 | -0.712 | -0.2360 | Yes |
| 35 | TXN |  |  | 8952 | -0.885 | -0.1760 | Yes |
| 36 | GPX4 |  |  | 8966 | -0.946 | -0.1014 | Yes |
| 37 | FTL |  |  | 8984 | -1.286 | -0.0000 | Yes |
Table: GSEA details [plain text format]

  

Fig 2: HALLMARK\_REACTIVE\_OXYGEN\_SPECIES\_PATHWAY: Random ES distribution      
 Gene set null distribution of ES for **HALLMARK\_REACTIVE\_OXYGEN\_SPECIES\_PATHWAY**
